# Adipokines, Oxidized Low-Density Lipoprotein, and C-Reactive Protein Levels in Lean, Overweight, and Obese Portuguese Patients with Type 2 Diabetes

**DOI:** 10.1155/2013/142097

**Published:** 2013-01-17

**Authors:** Maria João Neuparth, Jorge Brandão Proença, Alice Santos-Silva, Susana Coimbra

**Affiliations:** ^1^Centro de Investigação em Actividade Física, Saúde e Lazer, Universidade do Porto, Rua Dr. Plácido Costa 91, 4200-450 Porto, Portugal; ^2^Centro de Investigação das Tecnologias da Saúde (CITS), Instituto Politécnico da Saúde do Norte (IPSN), Cooperativa de Ensino Superior, Politécnico e Universitário (CESPU), Rua José António Vidal 81, 4760-409 Vila Nova de Famalicão, Portugal; ^3^Instituto Superior de Ciências da Saúde Norte (ISCS-N), CESPU, Rua Central de Gandra 1317, 4585-116 Gandra, Portugal; ^4^Laboratório de Bioquímica, Departamento de Ciências Biológicas, Faculdade de Farmácia, Universidade do Porto, Rua de Jorge Viterbo 228, 4050-313 Porto, Portugal; ^5^Instituto de Biologia Molecular e Celular (IBMC), Universidade do Porto, Rua do Campo Alegre 823, 4150-180 Porto, Portugal

## Abstract

*Aim*. Our aim was to study how different BMI scores may influence the levels of inflammation, oxidative stress, adipogenesis, glucose, and lipid metabolism, in lean, overweight, and obese Portuguese patients with type 2 diabetes mellitus (T2DM). *Methods*. We studied 28 lean, 38 overweight, and 17 obese patients with T2DM and 20 controls (gender and age matched). The circulating levels of oxLDL, CRP, and some adipokines—adiponectin, leptin, and chemerin—and the lipid profile were evaluated. *Results*. Obese patients presented significantly lower levels of adiponectin and higher leptin, oxLDL, and chemerin levels, as compared to the overweight, lean, and control groups. Overweight, compared to lean and control, subjects showed significantly lower adiponectin and higher leptin and chemerin levels; oxLDL values were significantly higher in overweight than in lean patients. Lean patients presented significantly higher chemerin values than the control. Obese patients presented significantly higher CRP values, as compared to lean patients and the control group. Obese and overweight patients presented significantly higher triglycerides values than lean patients. Except for CRP, all the observed significant changes between control and patients remained significant after statistical adjustment for the body mass index (BMI). *Conclusion*. The levels of leptin, adiponectin, oxLDL, CRP, and triglycerides in patients with T2DM seem to be more associated with obesity and less with diabetes. Chemerin levels were raised in lean, overweight, and obese patients, suggesting that, independently of BMI, an adipocyte dysfunction occurs. Moreover, chemerin may provide an important early biomarker of adipocyte dysfunction and a link between obesity and type 2 diabetes mellitus.

## 1. Introduction

Type 2 diabetes mellitus (T2DM) and obesity are independent global health problems, but an association between the two is known to exist. Patients with diabetes often present overweight and obesity [1]. Obesity prevalence is increasing significantly and obesity associates with the risk of T2DM and cardiovascular disease (CVD) events [2, 3].

The adipose tissue is a metabolically active organ, secreting numerous adipokines and proinflammatory cytokines, known to be important in the inflammatory and atherosclerotic processes [4]. Adiponectin has an anti-inflammatory activity and protects against metabolic and cardiovascular diseases [5]. Leptin is another adipokine and the reduction in its activity leads to severe insulin resistance and vascular dysfunction [6]. Chemerin is a novel adipokine that is associated with inflammation, adipogenesis, and glucose and lipid metabolism [7–9] all these adipokines are known to contribute to CVD events. Data concerning chemerin levels in prediabetics, in lean, overweight, and obese diabetic is not consensual [7, 10, 11]. 

Dyslipidemia and increased lipid oxidation are also associated with both T2DM and obesity. One of the major and early lipid peroxidation products is the oxidized low-density lipoprotein (oxLDL) [12], and the oxidation of LDL is considered a key event in the development and progression of atherosclerosis. OxLDL also damages the endothelium, promotes a chemotactic recruitment of the inflammatory cells and mediators through the vessel wall. 

Obesity is a proinflammatory state, usually presenting a low-grade chronic inflammation, with increased levels of proinflammatory cytokines and C-reactive protein (CRP), that are positively related to the body mass index (BMI) [13]. Inflammation has been associated with clinical progression of atherosclerotic disease and patients with high levels of CRP seem to exhibit an increased risk for an adverse cardiovascular outcome [14]. 

Our aim was to study how different BMI scores may influence in Portuguese patients previously diagnosed with T2DM the levels of inflammation, oxidative stress, adipogenesis, and glucose and lipid metabolism, by evaluating the circulating levels of oxLDL, CRP, and some adipokines—adiponectin, leptin, and chemerin—in lean, overweight, and obese Portuguese patients with type 2 diabetes.

## 2. Materials and Methods

### 2.1. Subjects

The protocol used was approved by the Committee on Ethics of the Instituto Superior das Ciências da Saúde Norte (CESPU), Gandra, Paredes, Portugal.

A group of 83 Portuguese adult patients with T2DM selected from the general population were enrolled in this study, after taking their informed consent. An interview with all subjects was performed, in order to collect clinical information and habits. The length of the disease was 9.2 ± 6.7 years old, and the age at time of diagnostic was 53.6 ± 9.8 years old (mean ± standard deviation (SD)). Patients had been diagnosed for a long time with T2DM and were all treated with oral hypoglycemic drugs. Patients were divided in 3 groups, according to BMI, lean (BMI ≤ 24.9 kg/m^2^; *n* = 28), overweight (BMI = 25.0–29.9 kg/m^2^; *n* = 38), and obese (BMI ≥ 30.0 kg/m^2^; *n* = 17) groups. The control group included 20 apparently healthy volunteers, age, gender, smoking, and alcohol drinking habits matched, with a BMI of 26.0 ± 2.5 kg/m^2^. Patients presenting inflammatory or infectious diseases and liver or kidney diseases were excluded from the study. Beside the oral hypoglycemic therapy in patients with diabetes, none of the patients nor the controls were receiving any medication that could interfere with our results (e.g., antioxidants, anti-inflammatory drugs, and antiobesity therapies). 

### 2.2. Assays

Blood from fasted (12 hours) subjects was collected into tubes without anticoagulant in order to obtain serum. None of the collected samples was icteric or hemolyzed.

Adipokines and oxLDL were evaluated by enzyme immunoassays (Human Adiponectin, R&D Systems, Minneapolis, MN USA; Leptin ELISA, Mercodia, Uppsala, Sweden; Human Chemerin ELISA, Biovendor Research and Diagnostic Products, Heidelberg, Germany; Oxidized LDL ELISA, Mercodia, Uppsala, Sweden). The serum levels of CRP were evaluated by immunoturbidimetry (Prestige 24i CRP Ultra, P.Z. Cormay, Lublin, Poland). The lipid profile (cholesterol, triglycerides, high-density lipoprotein cholesterol (HDLc), and low-density lipoprotein cholesterol) and glucose were evaluated by enzymatic colorimetric methods (Prestige, P.Z. Cormay, Lublin, Poland). To determine the levels of glycated hemoglobin, we used a spectrophotometric method (Prestige 24i HbA_1C_, P.Z. Cormay, Lublin, Poland). 

### 2.3. Statistical Analysis

We used the Statistical Package for Social Sciences (SPSS, version 17 for Windows, Chicago, IL, USA). A *P* value lower than 0.05 was considered as statistically significant. Comparisons between groups were performed using the Student's unpaired *t*-test or the Mann-Whitney *U* test, according to the Gaussian distribution of the substances. Measurements are expressed as mean ± SD or as median values (interquartile range), in accordance with the Gaussian distribution. Adjustment for confounding factors (BMI) was performed using analysis of covariance (ANCOVA), after transformation of variables (when necessary). 

As we were not able to study the control groups matched for BMI with our DMT2 patients, we performed statistical adjustment for BMI, using analysis of covariance (ANCOVA) (after transformation of variables, when necessary), in order to evaluate if the statistical differences observed between the control group and, T2DM groups were not associated with changes in BMI differences. 

## 3. Results

The clinical data for the control and for the lean, overweight, and obese patients with T2DM are presented at Table 1. The age, age at time of diagnosis, disease length, and gender, as well as smoking and alcohol drinking habits were similar for the 3 groups of patients. The glucose and glycated hemoglobin values (data not shown) did not present significantly different values for the three T2DM groups.

Concerning leptin, we found that for all the studied groups, control (*P* ≤ 0.01), lean (*P* ≤ 0.001), overweight (*P* ≤ 0.001), and obese (*P* ≤ 0.01) subjects, females presented significantly higher values than males. For the other studied parameters, no differences were observed according to gender.

**Figure 1:**
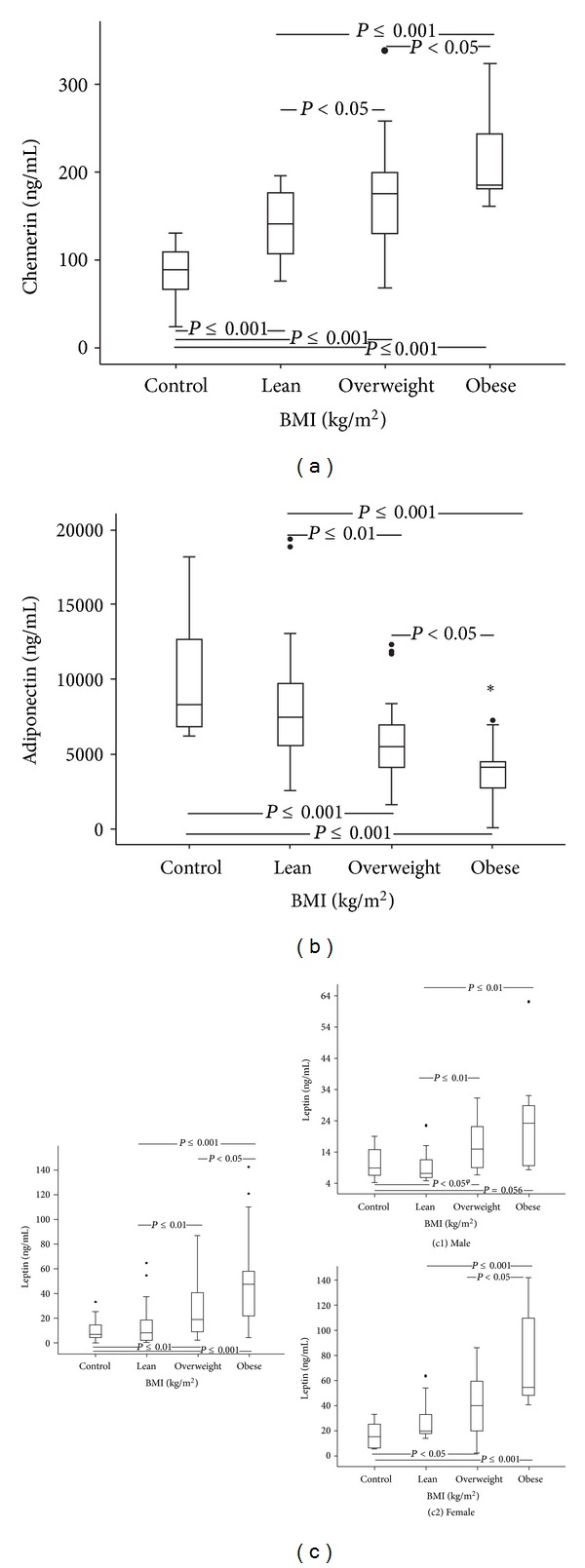
Values for control and for lean, overweight, and obese patients with type 2 diabetes mellitus of (a) chemerin, (b) adiponectin, and (c) leptin (^*ϕ*^lost of significance after adjustment for the body mass index (BMI); c1-leptin values for male subjects; c2-leptin values for female subjects).

Fifty-nine, 53, 39, and 60 percents of the obese, overweight, lean, and control subjects, respectively, were female. Considering only males (Figure 1(c1)), those who were obese presented significantly higher leptin levels than lean ones, and a trend towards higher levels as compared to the male controls; overweight males showed significantly higher leptin levels, as compared to lean and control males (that lost significance after adjustment for BMI). Considering the females (Figure 1(c2)), those who were obese presented significantly higher leptin levels than the overweight, lean, and control females (still significant after adjustment for BMI); the overweight females showed significantly higher leptin levels, as compared to the control females (still significant after BMI adjustment).

**Figure 2:**
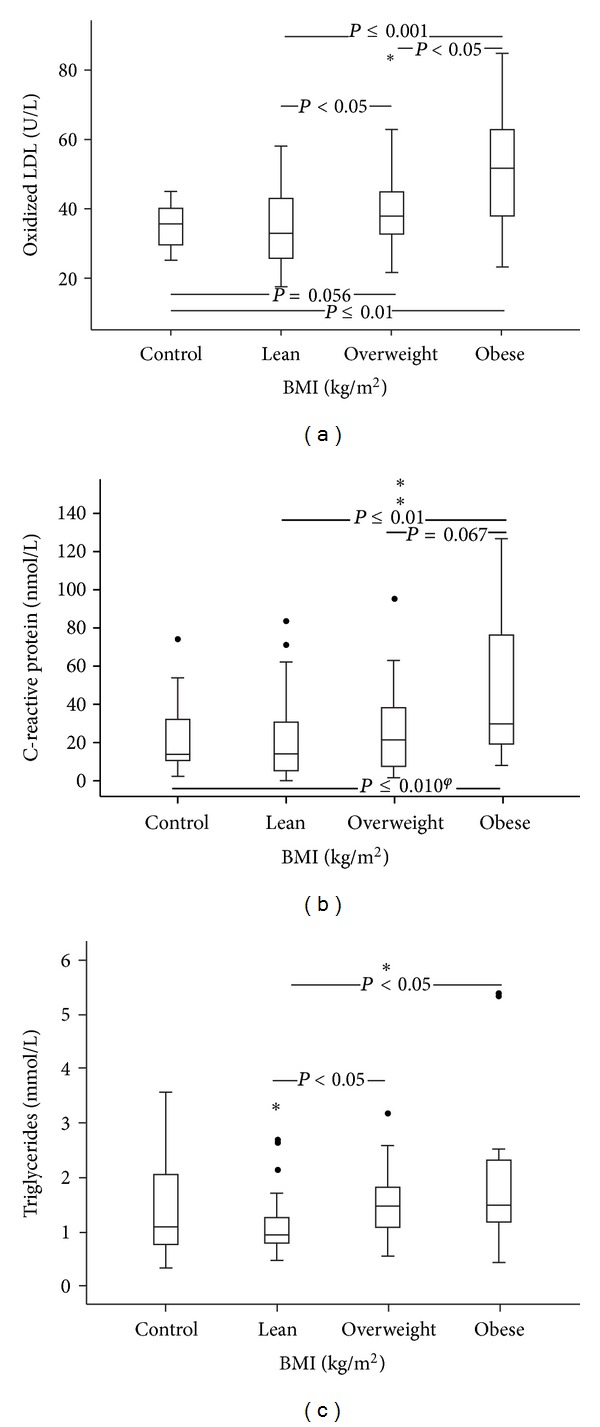
Values for control and for lean, overweight, and obese patients with type 2 diabetes mellitus of (a) oxidized low-density lipoprotein (oxidized LDL), (b) C-reactive protein, (c) triglycerides (^*ϕ*^lost of significance after adjustment for body mass index (BMI)).

The obese group (Figures 1(a)–1(c) and Figure 2(a)) presented significantly lower levels of adiponectin and higher levels of oxLDL, leptin, and chemerin, when compared to overweight, lean, and control groups (that were still significant after statistical adjustment for BMI). 

The overweight group showed significantly lower adiponectin and higher leptin and chemerin levels, as compared with lean and control groups (that were still significant after statistical adjustment for BMI); oxLDL concentration was significantly higher than that observed for the lean group and showed a trend towards higher values, as compared to the control (Figures 1(a)–1(c) and Figure 2(a)).

Lean patients presented significantly higher chemerin levels than the control (even after adjustment for BMI); no other significant changes were observed between lean patients and the control group.

Considering CRP (Figure 2(b)), the obese patients presented with significantly higher values than those presented by the lean and the control groups (that lost significance after adjustment for BMI) and a trend towards higher levels, when compared to the overweight group. For triglycerides (Figure 2(c)), the obese and overweight presented with significantly higher values than the lean group. We did not find any statistically significant differences between groups in the other studied parameters.

## 4. Discussion 

The aim of the study was to compare the circulating levels of oxLDL, CRP, and some adipokines in lean, overweight, and obese patients with T2DM, in order to analyze how these biomarkers vary with BMI and if any of them may track with insulin resistance, regardless of BMI. In several studies [15–21], the values of CRP, leptin, and adiponectin were evaluated in patients with T2DM, according to BMI. However, in these studies, patients were classified as lean and obese, and the overweight patients, when included in the study, were classified as lean; therefore, the changes associated to overweight were not evaluated, and, thus, the impact of a moderate increase in BMI was not evaluated. Considering that most of the patients with T2DM are overweight or obese, it would be important to study both groups. Moreover, most of the studies were not performed in Caucasian populations and it is known that there are differences in the adipose tissue distribution between ethnic groups [22]. As far as we know, the oxLDL levels were not compared between lean, overweight and obese T2DM patients. In opposition to our data, Bozaoglu et al. [10] did not find significant differences in chemerin levels between lean, overweight, and obese patients with T2DM.

 We found that the chemerin levels in lean, overweight, and obese patients with T2DM were significantly higher than the control. Indeed, lean T2DM subjects only differed from the controls for chemerin levels. Obese T2DM patients presented higher chemerin levels than overweight and lean patients; overweight T2DM patients showed significantly higher values, as compared to lean patients. 

Data concerning chemerin levels in diabetes is controversial. Some studies reported high chemerin levels in diabetes and obesity [23, 24] and that chemerin was correlated with BMI [7, 10]. As referred, Bozaoglu et al. [10] did not find significant differences between lean, overweight and obese Mexican-American patients with T2DM; nonetheless, in normal glucose-tolerant subjects, plasma chemerin levels were significantly associated with metabolic-syndrome-related parameters, including BMI, fasting serum insulin, triglycerides, and HDLc. The differences between their results and our data may arise from the different type of studied populations, as it is known that different ethnic groups present differences in adipose tissue distribution. Tönjes et al. [11] found that chemerin levels were already altered in prediabetic states, which is accordance with our data. It is known that chemerin has a crucial role in adipocyte differentiation and development, and that it may act as a modulator of different metabolic pathways in the mature adipocyte [9, 25]. Chemerin appears to act as modulator of the expression of adipocyte genes involved in glucose and lipid homeostasis [9]. Moreover, chemerin has been associated with several metabolic syndrome markers, namely BMI, triglycerides, blood pressure, and insulin resistance [10, 26]. Indeed, it was recently observed that insulin resistance seems to be a predictor of chemerin levels, independently of BMI [27]. In the present work, we found that chemerin levels were raised in lean, overweight, and obese patients, suggesting that in diabetes type 2, independently of BMI, an adipocyte dysfunction occurs. This change in chemerin levels was also observed in patients with T2DM, as well as in prediabetic states, suggesting that chemerin may play an important role in T2DM pathophysiology. Therefore, the study of chemerin may contribute to clarify the relationship between T2DM and obesity.

 Adiponectin has anti-inflammatory activity and protects against metabolic and cardiovascular diseases [5]. Leptin enhances the secretion of several cytokines and a reduction in its activity leads to insulin resistance [6, 28]. The levels of these two adipokines are known to be altered in obesity [15, 20, 29, 30]. Concerning adiponectin, an inhibitory feedback mechanism has been proposed, which would be triggered when adipose mass increases, probably as a consequence of an increased secretion of the other adipokines or of a reduced adipocyte metabolic function [31]. Regarding leptin, the resistance to its effects has been proposed to explain the reduced activity found in obesity [32]. We found that obese and overweight patients present significantly higher levels of leptin and lower levels of adiponectin, when compared with lean patients and with the control. Our data suggest that adiponectin and leptin levels in patients with T2DM are more associated with obesity and less with diabetes. Hansen et al. [21] studied Caucasian T2DM patients with BMI < 30 and BMI > 30 kg/m^2^ and found that patients with a BMI > 30 presented different values of adiponectin, when compared with patients with a BMI < 30 and with a control group. The authors did not find differences for leptin between groups, but it is known to exist a positive correlation between leptin and BMI [33]; no differences were found between BMI < 30 and the control group. These authors included lean and overweight patients with T2DM in the same group.

Concerning the lipid profile, as a result of an increasing adipose mass, overweight and obese T2DM patients presented higher triglycerides levels, when compared to lean patients. No differences were found for the other lipid parameters. However, concerning oxLDL, obese patients presented higher levels than any of the others groups; overweight patients showed higher values than lean patients and a trend towards higher values than the control group. An increase in LDL oxidation, as a consequence of increased oxidative stress and reduced antioxidant defenses, is a key event in the atherogenic process and is a known CVD risk factor. Our data suggests that, in overweight and obese patients with T2DM, the oxidant/antioxidant balance is altered, resulting in enhanced oxidative stress as reflected by an increase in oxLDL levels.

Obesity is a low-grade inflammatory state that associates with increased secretion of several proinflammatory cytokines, such as tumor necrosis factor-*α* and interleukin-6, which stimulate CRP production [4]. Our data show that increasing BMI is associated with an increase in inflammatory and oxidative stress markers, reflecting the relationship between obesity, inflammation, and oxidative stress. 

Overweight and obese patients, by presenting inflammation, raised LDL oxidation, and an altered adipokine secretion which have been associated with development of inflammatory and atherosclerotic processes and with insulin resistance, seem to be at a higher risk for CVD events. Patients with diabetes should be encouraged to reduce their adipose mass, through physical exercise practice, healthy diet habits, or, eventually, drug intervention, in order to reduce the risk for CVD events and to improve their quality of life. 

This study involves relatively small groups; further studies in a larger population are unwarranted in order to confirm our results.

## 5. Conclusion

In summary, the levels of leptin, adiponectin, oxLDL, CRP, and triglycerides in patients with T2DM seem to be more associated with obesity and less with diabetes. Chemerin levels were raised in lean, overweight, and obese patients, suggesting that, independently of BMI, an adipocyte dysfunction occurs. Moreover, chemerin may provide an important early biomarker of adipocyte dysfunction and a link between obesity and type 2 diabetes mellitus. 

## Figures and Tables

**Table 1:** Clinical data for control and for lean (body mass index, BMI ≤ 24.9 kg/m^2^), overweight (BMI = 25–29.9 kg/m^2^), and obese (BMI ≥ 30 kg/m^2^) patients with type 2 diabetes *mellitus* (T2DM).

	Control		T2DM patients	
	(*n* = 20)	BMI ≤ 24.9 kg/m^2^ (*n* = 28)	BMI = 25–29.9 kg/m^2^ (*n* = 38)	BMI ≥ 30 kg/m^2^ (*n* = 17)
Gender: F/M	12/8	11/17	20/18	10/7
Age (years)	60.0 ± 8.8	61.7 ± 10.0	63.6 ± 10.9	62.7 ± 9.4
Length of disease (years)	—	9.4 ± 7.4	9.4 ± 6.5	7.9 ± 5.9
Age at diagnosis (years)	—	52.3 ± 9.7	54.2 ± 10.1	54.7 ± 9.4
BMI (kg/m^2^)	26.0 ± 2.5	23.0 ± 1.7***	27.6 ± 1.3*	33.0 ± 2.6***

F: female; M: male.

*P versus control <0.050; ***P versus control ≤0.001.
